# Mental health professionals and key stakeholder views on the treatment and support needs of trauma and adult survivors of childhood sexual abuse in South Asia

**DOI:** 10.1371/journal.pmen.0000136

**Published:** 2024-09-13

**Authors:** Shivangi Talwar, Theodora Stefanidou, Helen Kennerley, Helen Killaspy, Rajesh Sagar, Rebecca Appleton, Jo Billings

**Affiliations:** 1 Division of Psychiatry, University College London, London, United Kingdom; 2 Oxford Cognitive Therapy Centre, Oxford Health NHS Foundation Trust, Warneford Hospital, Oxford, United Kingdom; 3 Department of Psychiatry, All India Institute of Medical Sciences, Delhi, India; 4 NIHR Mental Health Policy Research Unit, Division of Psychiatry, University College London, London, United Kingdom; University of Saskatchewan, CANADA

## Abstract

People who have experienced trauma, especially adult survivors of childhood sexual abuse (CSA) are at risk of experiencing subsequent emotional and mental health difficulties. South Asian countries have high prevalence rates of CSA and other forms of complex trauma. Despite the requirement for mental health support for trauma survivors in South Asia, their needs are poorly understood, and specific interventions are still in their infancy. We aimed to explore the views of mental health professionals and key stakeholders on the mental health and support needs of trauma survivors, especially CSA survivors in South Asia and the treatment and support available. We interviewed mental health professionals and key stakeholders from six countries in South Asia who had experience working with trauma survivors. Data were analysed using reflexive thematic analysis. We interviewed 17 professionals and key stakeholders working in Afghanistan (n = 1), Bangladesh (n = 2), India (n = 8), Nepal (n = 2), Pakistan (n = 2) and Sri Lanka (n = 2). Four main themes were developed: mental health symptoms as the tip of the iceberg; a pragmatic approach to treatment and services; systemic factors are roadblocks to trauma services; cultural beliefs and practices across communities. We found that the participants view survivors’ difficulties as a combination of mental health problems and relationship difficulties, often dominating the mass of trauma. Despite systemic barriers, they extended their services to the survivors to make them as applicable as possible to the clients’ needs. For this, they considered the clients’ context. Overall, these professionals in South Asia acknowledged that the treatment and support that should be provided to trauma survivors, specifically adult CSA survivors, have not yet been formally established in South Asian contexts. Future research is needed to inform guidance for specific communities in the South Asian context.

## Introduction

Survivors of childhood sexual abuse (CSA) are at risk of experiencing difficulties with mental health and relationships in their adulthood. Experiencing CSA is seldom a one-off incident and usually includes multiple exposures to trauma [1]. Adverse events such as CSA may result in post-traumatic stress disorder (PTSD) symptoms, such as recurrent ‘reliving’ of the event(s), avoidance of reminders of it, and feeling under current threat even when no specific threat is present [2]. The harm to the individual’s sense of trust caused by CSA may also impede the development of secure attachments in later life [2] possibly contributing to the ‘complex’ symptoms within Complex Post Traumatic Stress Disorder (CPTSD). These complications, which occur in addition to PTSD symptoms [2] include difficulties in affect regulation and interpersonal relationships, decreased self-esteem, and feelings of shame, guilt or failure [2].

Asia has the second highest prevalence of reported CSA globally ranging between 3.3–58% [3] The South of Asia is the most populated region within Asia. It includes India, Bangladesh, Pakistan, Sri Lanka, Afghanistan, Bhutan, Maldives and Nepal [4] High prevalence rates of CSA have been reported across all South Asian countries, except Afghanistan where prevalence studies have not yet been conducted [5]. Under-reporting due to reasons such as poor understanding of CSA and stigma associated with disclosure of abuse [6, 7] mean that the prevalence may be considerably higher.

Common to the South Asian countries is a collectivist culture and ideologies about family such as maintaining family reputation [8]. Nevertheless, all South Asian countries differ from each other in terms of structural differences in mental health services and varied legal, religious, socioeconomic and political conditions [9]. India, Pakistan and Bangladesh have established mental health acts and ordinances [10–12]. However, Bhutan, the Maldives and Afghanistan do not have specific mental health legislation, but they do have national mental health programmes, policies and strategies [13–15] Nepal and Sri Lanka have mental health laws and policies in draft form awaiting approval [16, 17].

Despite PTSD prevalence of 17.2% in South Asia [18] there is an absence of specific treatment guidance for PTSD/CPTSD in South Asia [5]. The recommended first line treatments for PTSD in the United Kingdom (UK) are Trauma-focused Cognitive Behavioural Therapy (TF-CBT) and Eye Movement Desensitization and Reprocessing (EMDR) [19], targeting trauma-related memories in PTSD which is therapeutically more beneficial than targeting post-traumatic symptoms [20]. For adults diagnosed with PTSD, sometimes additional pharmacological treatments for the symptoms of PTSD or for co-occurring mental health conditions could include antidepressants and other psychotropic medications [19]. Treatments targeting the additional complex symptoms of CPTSD have limited evidence, but the NICE Guideline recommendations include increased duration or number of psychological intervention sessions, managing other difficulties, like interpersonal problems or substance misuse, and discussing plans for managing after therapy has terminated [19]. Although the guidance for the clinical management of PTSD/CPTSD has a growing evidence base, adult survivors of CSA may have further support needs outside the purview of a clinic such as social support [21].

The lack of evidence based trauma-focused treatments in South Asia does not necessarily reflect a lack of quality services or good practice for people affected by trauma. The developing mental health laws and policies in some of the South Asian countries (India, Bangladesh, Pakistan and Sri Lanka) are undergoing a transition from colonialised training programmes for mental health professionals to a more community tailored model, although this transformation is still under scrutiny [22]. For now, however, this is likely to further restrict the services for specific populations like adult CSA survivors who may require access to specialist services and professional expertise.

In the present study, we aimed to explore the views and experiences of mental health professionals (MHPs) and other key stakeholders in South Asia about current treatments for people affected by trauma with a specific focus on adult survivors of CSA. Specifically, we wanted to understand practitioners’ and stakeholders’ current approaches to treating adult survivors of CSA, what they perceived survivors’ treatment needs to be, and what barriers they experienced in meeting these needs. We decided to focus on trauma more broadly because CSA is under-detected/under-reported in South Asia. Therefore, we recruited professionals with expertise in treating trauma more broadly and then probed about what might be important additionally in the treatment of adult survivors of CSA. Adult CSA survivors are likely to have experienced other types of trauma and abuse. Further, in a South Asian context, many professionals are working with survivors experiencing financial, military, disasters and political-related complications. For these reasons, we think it would be negligent to view CSA in isolation.

## Methodology

### Ethics statement

The study was approved by the UCL Research Ethics Committee (Ethics ID 22535/002). A formal written consent was obtained from all the participants. We followed the Standards for Reporting Qualitative Research [23] in this paper.

### Study design and population

We have employed an exploratory qualitative study design to examine the views of mental health professionals and key stakeholders in South Asia. For this, we included mental health professionals who had qualified in a South Asian country, were of South Asian origin, residing in South Asia, at least 25 years of age (to complete qualifications), with at least two years of clinical and/or applied clinical research experience in a South Asian country. Other key stakeholders were eligible to take part if they were third-sector workers of South Asian origin, working specifically with people with CSA experiences in a South Asian country, at least 18 years of age, and with at least two years of work experience.

### Recruitment of participants

We recruited participants purposively through LinkedIn and other professional networks of the research team members between 1^st^ January and 31^st^ August 2023. Participants were recruited from different regions and cultural backgrounds, at different career stages and working in varied settings, to invite variation in views and perspectives. We aimed for 8–15 participants to balance the anticipated challenges of recruiting professionals across South Asia. The first author, ST, provided a brief overview of the study to those who expressed an initial interest in the study and then followed up with an email explaining the study aims and attaching the participant information sheet. Those who were willing to participate then completed an online written informed consent form before they were interviewed which was then stored on OneDrive on a password protected computer.

### Data collection

Semi-structured interviews were led by the first author, ST, on online platforms such as MS Teams, Zoom or Google Meet, depending on what was most accessible to the participant. The interviews were conducted in English and Hindi by ST who is fluent in both languages. A topic guide was used to steer the interviews; this was developed by ST, JB and RA (S1 and S2 Text). The questions enquired about the perceived needs of adult survivors of CSA and current treatments or support programmes available in the participant’s setting/organisation and country. Further, the interviewer asked participants for suggestions for cultural modifications in delivering those treatments and programmes.

The interviews were audio recorded through the video calling platform and transcribed using MS Stream. Any potentially identifiable information was deleted from the transcripts. Three transcripts in the early phases of data collection were reviewed by RA to assess whether the interviews were in line with the study objectives and to guide ST on the ongoing data collection process. To ensure retention of the meaning of data, three transcripts in Hindi were not translated to English, but any minor Hindi/Urdu phrases in the remaining predominantly English transcripts coded by TS and RA were translated.

### Data analysis

We analysed the data using an inductive and iterative approach following the principles of reflexive thematic analysis [24]. We used MS Excel and MS Word throughout the analysis. Using an inductive approach, the first author (ST) led the coding, and all the coders immersed themselves in the data. This process was enhanced by using multiple coders wherein ST coded all the transcripts, TS coded four, and RA coded one of them.. ST, TS and RA agreed collaboratively on a the coding approach [24]. ST maintained a reflexive journal during data collection and coding to enhance the trustworthiness of the study and improve the quality of analysis [25]. Preliminary findings were also presented and discussed at a meeting with the other team members who are clinical academics and peer academic researchers. ST, RA and TS met separately to discuss provisional candidate themes identified from the data. ST, RA, JB and TS reviewed the candidate themes and agreed on the final themes and subthemes. The theme and subtheme names were refined to best capture the participants’ experiences. All authors reviewed the final analysis. A summary of the findings was subsequently sent to all the participants to inform them about the study findings and dissemination plan.

### Quality and rigour

Our analysis followed an interpretative reflexive process wherein only one person collected the data, but three other members were closely involved during the analysis. Wider team were invited to contribute to the conceptualisation of the study design, reviewing and refining of the themes, and drafting of the manuscript.

We used a critical realist ontological position and a contextual epistemological position throughout the study. We chose this philosophical orientation to understand the views and experiences of our participants as connected by a common thread but also as individuals. This approach also enabled us to reflect on the data as clinicians and researchers. A critical realist orientation led to interpretation of the data beyond description.

Using maximum variation sampling [26], we aimed to recruit participants from different regions, from varied cultural and educational backgrounds and genders. We also situated the data within the context of the professional and their clients. This approach enabled us to facilitate transferability and demonstrate sensitivity of the context [27].

We collected the data using videoconferencing softwares to ensure that we were able to recruit participants from diverse backgrounds and regions. We acknowledge that this could have affected the participant-researcher relationship, however, this methodology enabled us to access a geographically wide group of participants with minimal participant burden.

### Researcher characteristics and reflexivity

The research team comprised academic researchers and clinical academics in the UK and India. Table 1 describes the diversity and strength of all the authors informing the credibility of the results. All authors have mental health academic backgrounds with experience conducting research or working clinically with trauma-affected people and/or undertaking qualitative research. Our cultural backgrounds and professional experiences of working with people affected by trauma and/or working in South Asia (India) or people of South Asian origin informed the study design, topic guides and analysis.

**Table 1:** Characteristics of team members.

Initials	Researcher’s background
ST	Indian, female, clinical psychologist in India; Interested in applied clinical research with trauma-affected populations, specifically adult survivors of CSA
	During data collection, situating ST as the interviewer within the network of South Asian professionals facilitated ground-level discussions. ST received training and supervision from JB and RA, expert qualitative researchers.
TS	White, Greek, female, mixed methods researcher; interest in loneliness; previous experience in conducting research on sexual violence and mental health research
HKe	White, British, female, consultant clinical psychologist in the UK; specialising in research and clinical work with adult CSA survivors
HKi	White, British, female, senior consultant psychiatrist and clinical academic; working in the fields of rehabilitation psychiatry and the development and evaluation of complex interventions for people with complex psychosis in the UK
RS	Indian, male, psychiatrist practicing in India; interested in the mental health of children and adolescents with a specific focus on child sexual abuse and learning difficulties
RA	White, British, female, senior researcher; interest in child and adolescent mental health and expertise in mental health policy and qualitative research
JB	White, British, female, consultant clinical psychologist in the UK; interested in clinical work and applied clinical research with trauma affected populations

## Results

A total of 17 professionals working in South Asian countries participated in our study. Of those, 15 were mental health professionals; six were from India, one from Afghanistan and two each from Pakistan, Nepal, Sri Lanka and Bangladesh. The two remaining participants were stakeholders (an organisation lead and a programme manager) working with CSA survivors in India. Despite efforts to recruit participants from Maldives and Bhutan, we did not receive responses from the professionals contacted. All participants were within the age range of 26–50 years with a mean age of 34.65 years. Their years of experience, including their training periods, ranged from 2 years 10 months to 27 years, averaging at 9.76 years. Some of the clinicians had dual roles, including clinical work and academic teaching or research. All participants had completed at least a postgraduate level of education. Further participant characteristics have been provided in Table 2.

**Table 2:** Characteristics of participants.

Characteristic of participants (n = 17)	N (%)
**Mental health professionals** *	**15 (88.23)**
Clinical Psychologist	11 (64.70)
Psychologist	2 (11.76)
Psychiatrist	2 (11.76)
*Lecturer/Teacher*	*6 (35*.*29)*
*Researcher*	*2 (11*.*76)*
**Stakeholders**	**2 (11.76)**
Manager at a non-governmental organisation	1 (5.88)
Lead of a non-governmental organisation	1 (5.88)
**Age of participants**	
25–30	5 (29.41)
31–35	6 (35.29)
36–40	4 (28.57)
45–50	2 (11.76)
**Highest degree completed**	
MPhil Clinical Psychology/Psychology	6 (35.29)
PhD Clinical Psychology/Psychiatry	5 (29.41)
MA/MSc Psychology/Clinical Psychology/Forensic Psychology	4 (28.57)
MD Psychiatry	2 (11.76)
**Countries of Residence and practice**	
India	8 (47.05)
*North*	*4 (28*.*57)*
*South*	*1 (5*.*88)*
*Northeast*	*2 (11*.*76)*
*West*	*1 (5*.*88)*
Sri Lanka *(Southwest)*	2 (11.76)
Afghanistan *(East)*	1 *(5*.*88)*
Nepal *(North)*	2 (11.76)
Bangladesh *(Southeast and Central)*	2 (11.76)
Pakistan *(South)*	2 (11.76)
**Years of work experience**	
More than 10 years	8 (47.05)
Up to 10 years	9 (52.94)
**Gender of participants**	
Female	12 (70.88)
Male	4 *(28*.*57)*
Gender fluid	1*(5*.*88)*

Note *Participants may have held dual professional roles.

The interviews lasted between 41 minutes to 99 minutes and were an average of 63 minutes long.

Table 3 presents a list of the themes and sub-themes developed. These are discussed in detail below with anonymized participant quotations:

**Table 3:** Developed themes and sub-themes.

**1. Mental health symptoms as the tip of the iceberg**
1.1 Disguised or undisclosed trauma
1.2 That’s not trauma, is it?
1.3 To acknowledge or not to acknowledge the past?
**2. Pragmatic approach to treatment and services**
2.1 Quick fixing
2.2 Bridging the gap between theory and client needs
2.3 Engaging other professionals and families
**3. Systemic factors are often roadblocks to trauma services**
3.1 Helplessness due to lack of means
3.2 Non-specialised services and lack of regulations
3.3 Supporting us to support them
3.4 Social, economic and political constraints
**4. Cultural beliefs and practices across communities**

### 1. Mental health symptoms as the tip of the iceberg

In our study, the professionals and stakeholders described that the mental health difficulties experienced by their survivor clients are often reported and are visible on the surface. Beneath the surface, the mass of the iceberg are their traumatic experiences.

#### Disguised or undisclosed trauma

All the mental health professionals interviewed suggested that most trauma survivors did not associate their current difficulties with their recent or past traumatic experiences. They reported that trauma survivors in South Asia commonly presented with difficulties related to interpersonal relationships, school and workplace, symptoms of common mental disorders (CMDs) and severe mental illnesses.

*“feeling low or feeling anxious, feeling insecure…anxiety symptoms like shortness of breath or like not feeling able to sleep properly, decreased appetite, different physical, biological symptoms…behavioral issues, like excessive mobile watching…”*

*-Academic and Clinical Psychologist in Nepal*

Mental health professionals in Pakistan, Nepal, Sri Lanka and some parts of India specified that their clients presented with trauma of an interpersonal nature more often than collective trauma (i.e. shared by a group such as war and disaster). Some clinicians from India reflected that people who experienced traumatic experiences in adult life were more likely to have an established support system and a greater sense of self, unlike those experiencing trauma in childhood. Some clinicians from India and Nepal reported that they hypothesized a history of CSA or trauma histories in their clients with a personality disorder, especially borderline type.

*“…may be having some like for example, borderline personality behavior issues. Yeah, they are mostly with traumas.”*

*-Academic and Clinical Psychologist in Nepal*

All mental health professionals, especially Psychologists, reported that sometimes adult CSA survivors would consult them on the suggestion of other medical professionals. According to these professionals, clients who were adult CSA survivors rarely consulted these clinicians for help in managing traumatic memories of their CSA experiences.

*“…….who have been told by their psychiatrist that…looks like in your history, this is because also of something that you have gone through in the past. So you should consult a psychologist… .they come to me asking question that do you think that has links to the problem that I am experiencing now because my psychiatrist says that now I should see you…but they do not exactly understand why we are doing this, why we have to talk about something which is gone in the past.”*

*- Clinical Psychologist in North India*

The stakeholders included in our study had survivors contacting their organisations to directly seek help or discreetly seek more information by saying they were a survivor’s friend or family member.

Overall, there was a pattern of survivors avoiding discussion about developmental sexual trauma. After a few sessions, survivors sometimes disclosed or touched upon their CSA experiences, with or without realising that those experiences may be linked to their current symptoms.

*“…when we take their histories and go in depth… .after a few sessions and depending on our questions and how resistant the client is… .if the client is comfortable by then and…does not feel threatened…so we avoid asking such questions in initial sessions…”*

*-Academic and Clinical Psychologist in Pakistan*

#### 1.2 That’s not trauma, is it?

Mental health professionals from Sri Lanka, Nepal, Afghanistan and India said that their clients tended to construe CSA as abusive but not other forms of child abuse or neglect experiences.

*“…here, physical abuse is as common as boxes of sweets. They don’t want to consider physical abuse as abuse. It’s considered as a part of parenting and I openly say that abuse is happening in the name of parenting…”*

*-Clinical Psychologist in North India*

Some professionals also suggested that years of catastrophic events in their countries/regions have made people “resilient” resulting in them not labelling some critical incidents as traumatic.

*“…you know we’ve had so many…traumatic events, disaster…But I think there’s a high resilience of the people. Maybe because we are, umm, there’s some sort of desensitisation… perhaps also due to the collectivist nature of our societies… ..after the tsunami…NGOs…came down expecting to treat trauma…people weren’t as traumatized as they anticipated.”*

*-Academic and Clinical Psychologist in Sri Lanka*

Catastrophic or not, some professionals in Bangladesh, Pakistan and India explained that those who experienced ‘traumatic’ situations may have more pressing issues unrelated to their mental health, with traumatic experiences taking a backseat.

#### To acknowledge or not to acknowledge the past?

Some of the professionals in our sample did not directly ask clients if they had experienced trauma, such as CSA. Some professionals included processing trauma memories as one of the main goals of treatment. Other professionals assessed whether targeting traumatic experiences like CSA would benefit survivors or not and made treatment decisions accordingly.

Some mental health professionals from India, Afghanistan and Nepal stressed that treatments and services focused on traumatic memories were seen to be unpleasant and therefore should be avoided. An Indian participant specified that survivors’ family members would discourage such discussions.

*“And what I generally see is after 2–3 sessions, they are like oh this session didn’t go happy. You know I’m not happy because maybe you touched upon certain things which I didn’t want to……”*

*-Clinical Psychologist in North India*

### 2. Pragmatic approach to treatment and services

Our participants highlighted that they usually needed to deter from following a structured, standardised approach with their survivor clients. They tended to be pragmatic considering the limited availability of resources and diverse needs of their clients.

#### 2.1 Quick fixing

Most professionals, clinicians and stakeholders alike, said that trauma survivors want a ‘quick fix’ for their mental health, relationship and other difficulties instead of processing their trauma memories.

*"mostly they are, the thing is they are looking for quick fix and medicine is the quick fix here”*

*-Clinical Psychologist and PhD candidate in Northeast India*

According to most professionals, the feasibility of the survivors engaging in long-term treatment was a challenge. Hence, their clients would request medication and immediate support rather than multiple consultations.

### 2.2 Bridging the gap between theory and client needs

Most professionals recognised that there was a gap between the published evidence developed in non-South Asian countries and the clinical presentation of trauma survivors in South Asia. Most mental health professionals explained that they adhere to the current non-South Asian evidence but adapt what would be required for their client. These adaptations were made according to survivors’ education level, region and mental health literacy. However, even after they made adaptations, personalising/tailoring treatments presented a professional challenge.

*“Clinicians just sort of, umm, take the fundamental concepts and then without necessarily subjecting it to scientific study, that culture adapting in how they’re delivering for the individual clients…”*

*-Academic and Clinical Psychologist in Sri Lanka*

Mental health professionals accepted that their informal adaptations were not validated in their cultures, and there is a need to formally establish the evidence base for treatments and services for trauma-affected people, including adult CSA survivors.

*“I think we when we try to imitate or try to practice the way they (in the West) are, I think sometimes the things do not fit at that time (in our country).”*

*-Academic and Clinical Psychologist in Nepal*

#### 2.3 Engaging other professionals and families

Some professionals based in Nepal and Sri Lanka suggested a multidisciplinary team for meeting the mental health needs of adult CSA survivors.

*“…in a team yeah, there is a psychiatrist and clinical psychologist…sometimes there are different forensic teams also when we explore about these things…includes forensic expert, ya, medicine physicians, ya, forensic expert and some psychosocial trained counselors.”*

*-Psychologist in Sri Lanka*

Psychiatrists in Afghanistan and Nepal emphasised the role of psychologists in treating trauma-affected people, including adult CSA survivors. One Psychiatrist highlighted that they may diagnose and prescribe medications for all their patients alike, but their Psychologist colleagues have to plan the treatment according to the client’s difficulties. Psychologists predominantly promoted the importance of psychological therapies for treating trauma-affected people, including CSA survivors. Whilst some psychologists also mentioned delivering the same treatments to all trauma-affected people, others said that they would offer different treatments to survivors of childhood trauma and to those who suffered acute trauma in adulthood.

*“So I would think that approach might be, the approach of therapeutic frameworks might be similar but it would be according to individual.”*

*-Psychologist in Sri Lanka*

Psychologists in our sample treated trauma survivors using a range of treatment approaches including EMDR, Acceptance and Commitment Therapy (ACT), Dialectical Behaviour Therapy (DBT), psychodynamic approach (for eg. Object Relations framework) and TF-CBT. However, most psychologists preferred using CBT. Some psychologists from India, Nepal and Bangladesh mentioned pharmacological treatment alongside psychological therapies. The Nepalese clinicians further appreciated ‘one stop centres’ in their country for people affected by violence and abuse. These comprised a team of counselors and psychologists with additional legal and medical support.

*“…some of the clients were referred by One Stop Crisis Management Center… .that works mainly gender based violence…among them, I think most of them, they are, yeah, having different kinds of trauma, intimate partner violence or childhood sexual abuse.”*

*-Academic and Psychiatrist in Nepal*

Stakeholders, working only with CSA survivors, said that they encourage the survivors to engage in their organisation’s programmes and support other survivors. The programmes included support groups, mental health support, referrals to clinicians and awareness initiatives. These stakeholders suggested that seeking help from mental health professionals within and outside their organisation was crucial.

All professionals highlighted the importance of family, either including them in the client’s treatment or creating boundaries with the family members.

*“when nobody in the family knows that you have been abused by this person. But being expected to be part of family functions and to be part of, you know, and retain your sanity, for example is very much very Indian, South Asian…whether you are living with them, you are never away from them in a way…but relationship with the abuser…like your father’s 4th cousin is also a family member…”*

*-Stakeholder in North India*

### 3. Systemic factors are often roadblocks to trauma services

According to our participants, the mental health, healthcare and wider national-level systems hinder offering the treatments and services for trauma-affected people, specifically CSA survivors.

#### 3.1 Helplessness due to lack of means

Participants across South Asian countries highlighted that they do not have the resources to improve the trauma services in their countries. All professionals thought that the mental health and social workforce in their country is insufficient to meet the needs of high numbers of trauma affected people in their clinics and organisations. Some Indian, Sri Lankan and Nepalese clinicians expressed feeling overburdened with the high footfall of trauma affected people requiring treatment.

*“…in our states there are not much of a mental health professional, especially clinical psychologist, so there is lots of demands… ..So besides my clinical hours, I am used to have a patient refer from the doctors, psychiatrist… .”*

*-Clinical Psychologist in Northeast India*

Linked to the mismatch between the number of service providers and users, mental health professionals in our sample spoke about the difficulties in offering personalised care to clients affected by trauma.

Some professionals in India, Sri Lanka, Bangladesh and Nepal suggested that both service provision and research with trauma-affected people needed to be improved by increasing allocated funds. A stakeholder in India mentioned that they are eager to improve published research with adult CSA survivors but lack sources to secure funding and facilitate such studies.

*“We just don’t have people who are willing to do research with us and get us some money to do it. So it’s always part of our wish as a way of putting out work.”*

*-Stakeholder in North India*

#### 3.2 Non-specialised services and lack of regulations

Most mental health professionals from Sri Lanka, Nepal, Bangladesh and Afghanistan expressed dissatisfaction with the clinical training facilities for mental health professionals in their countries. Their disappointment was not limited to the breadth of training but also included the number of training institutes and intake of trainees. A few of them had received specialist training from other South Asian and non-South Asian countries to upskill themselves.

*“In Bangladesh, we don’t…have people… .who specialized in this field, so…there should be trainings or…currently we don’t have… .enough opportunities… if you…want to specialize on these issues, you need to go other countries… .”*

*-Clinical Psychologist and researcher in Bangladesh*

Some clinicians from India, Nepal, Bangladesh and Sri Lanka expressed feeling unequipped to offer trauma therapy, more so for special populations like adult CSA survivors.

One stakeholder who was working with adult CSA survivors in India highlighted that their organisation had limited resources, and it was a challenge to find a suitable clinician for survivors contacting their organisation.

Mental health professionals in Bangladesh, Pakistan, Nepal and Sri Lanka explained that the lack of a regulatory/statutory body and still developing mental health laws and policies have been impacting their clinical practice and further training.

*" in Sri Lanka is that we don’t yet have an established Sri Lanka psychological association. It’s sort of unofficial, it’s not yet recognized from the parliament as a statutory body, so there’s no way of controlling the fear of…so called therapists or counsellors and you know, education and qualifications.."*

*-Academic and Clinical Psychologist in Sri Lanka*

#### 3.3 Supporting us to support them

Some professionals in India and Sri Lanka spoke about the vicarious trauma experienced by them and their colleagues and. recommended specialised support for clinicians and researchers working with this population. The key stakeholders working with CSA survivors in India reported that their organisations have special services for disclosures of CSA experiences by the employees and support for exposure to client disclosure. Most of these professionals, clinicians and stakeholders, conveyed that trauma practitioners and researchers, without access to mental health support, will find it difficult to continue providing services.

*"I do feel that mental health professionals working with vulnerable population, they do require, some support in that way, but especially those working with trauma… .one of my close friend… .tells me about the numbness that she feels… .an emotional responsiveness to situation has reduced because she has heard so much… "*

*- Clinical psychologist in South India*

### 3.4 Social, economic and political constraints

Some mental health professionals from Afghanistan, India, Sri Lanka, Pakistan and Bangladesh expressed their concerns about the inability of their clients to afford multidisciplinary professional help and/or long-term treatment and support.

*“… .they cannot pay for psychologists or they cannot pay two fee for doctors and psychologist as well.”*

*-Psychiatrist in Afghanistan*

Pakistani, Sri Lankan and Afghan professionals further elaborated on how the financial difficulties experienced by their countries, as a whole, have led to traumatic conditions. Clinicians from Afghanistan, Sri Lanka, Pakistan and some parts of India highlighted the impact of political conditions on the traumatization of their clients.

*“…children here in Sri Lanka can be quite vulnerable to childhood traumas..but fact that in a developing country…mothers from the…lower socioeconomic strata leaving the country to work as housemaids in the Middle East. So leaving a lot of children population quite vulnerable…”*

*-Academic and Clinical Psychologist in Sri Lanka*

### 4. Cultural beliefs and practices across communities

Our participants reported that working with trauma survivors, specifically CSA survivors, was impacted by cultural backgrounds, religious beliefs and community practices. They needed to take these factors into consideration to engage and plan the treatment.

Most mental health professionals stated that CSA survivors form an understanding of their trauma and CSA experiences in line with their cultural norms and mental and sexual health literacy. According to them, this personalised understanding could deter survivors from seeking professional help and lead to dropout.

*“So depression, what is written in ICD-10 or 11 or DSM 5 might not be relevant to what we (in Bangladesh) understand about depression.”*

*-Clinical Psychologist and researcher in Bangladesh*

Some clinicians from all the included countries suggested that following a less structured therapy process and including the family in treatment were important with their trauma-affected clients. Providing sexual health information in psychoeducation was a specific suggestion for adult CSA survivors. This was unlike what they had read in non-South Asian published evidence.

Specific to the northeast region of India, some clinicians spoke about the trauma related to military and political unrest and natural disasters precipitating and perpetuating mental health conditions. An Indian clinician practising in a northeast Indian city explained that middle-aged and older adults have traumatic memories of military conflicts experienced by their community within their state. Another clinician from a different northeast Indian state explained the traumatising impact on generations of locals due to annual floods and national registration.

A Pakistani clinician highlighted that adult CSA survivors who continue to experience abuse into adulthood (particularly with same-sex perpetrators) experience this as contravening their religious codes, which makes it harder for them to speak out.

*“This is crime if we talk about Islam to have male-to-male relationship plus extramarital affair, be it bisexual…client considered it a huge crime…”*

*-Academic and Clinical Psychologist in Pakistan*

An Afghan clinician mentioned how faith healing may take precedence over mental health support.

*“…After some time when the patient became fine and got improvement, some of the colleagues of her mother said leave the treatment, please take your daughter to mulla (Muslim clergy)…they give some taaveez (religion pendant or bracelet to wear)…She left the treatment after 3–4 months. She came again… .I said why she got bad or worse from previous months. The mother cried…I left treatment.”*

*-Psychiatrist in Afghanistan*

According to a clinician in Mizoram (a state in India with predominantly Christians having tribal ancestry), mindfulness and yoga have roots in other religions, and it would not be acceptable for survivors in Mizoram to practice these approaches. Hence, this clinician in Mizoram had to explain the rationale for mindfulness practices and modify them to accommodate the needs of their clients. A Nepalese clinician with work experience in Nepal and India highlighted how the excessive engagement of family members of their clients in India and Nepal are alike. A Bangladeshi clinician suggested the need for an “indigenous” approach to treating trauma survivors. Another clinician from Afghanistan spoke about the relevance of male family members in the treatment of female clients.

*“…the doctor first convinced…husband or the brothers that we need to talk with this female alone. After that if he permit doctor can speak with female patients otherwise he say no doctor that is not allowed. Not important…she has no any problem with me…Everything is clear with us…In this case I say that 90% female say no no brother or no husband or son, I I dont have any problem but maybe 10% of female can talk with doctor alone in the room…”*

*-Psychiatrist in Afghanistan*

## Discussion

We aimed to explore the views of mental health professionals and key stakeholders from South Asia about the needs and current treatments for people affected by trauma. Specifically, we wanted to understand mental health professionals’ and other stakeholders’ perceptions of the mental health and support needs of adult CSA survivors, what approaches they use to treat and support them, and what difficulties professionals and stakeholders need to overcome to meet survivors’ needs.

Overall, the professionals and stakeholders in South Asian countries highlighted psychological therapies, pharmacological treatments, support groups and social initiatives as instrumental in supporting trauma survivors, specifically CSA survivors. Elaborating on these modalities of support, they highlighted that the systemic factors impacting the treatment, help-seeking and service provision within their regions and countries. They have observed traumatization appearing in their clients in concealed forms, requiring prioritising the management of the presenting symptoms. The professionals and key stakeholders raised a question regarding the meaning of trauma in South Asia, and whether there is a need to address and process it. A key finding of this study was professionals’ attention to the incongruity between the needs of their clients and the current services, support and theory available. They voiced the need for adapting treatments in their countries once an indigenous understanding of trauma has emerged. Multidisciplinary management and time-bound support could improve the services offered.

### Survivors’ initial presentation, expectations and reservations

This study’s findings suggest that trauma survivors in South Asia tend to disclose their traumatic experiences as the sessions progress. Disclosure of interpersonal traumatic experiences such as CSA is more stigmatised than violent and other traumatic incidents, for example, related to work [28]. Adult CSA survivors may struggle to disclose to mental health professionals due to issues like anticipation of disbelief [29]. In non-western contexts, religious and spiritual beliefs can impact the reporting of trauma [30]. Specifically in South Asian countries, collectivistic cultures are often linked to social integration, which could further limit the disclosure of interpersonal trauma [28]. Speaking about psychological trauma as an outcome of traumatic events may take a backseat due to anticipated shame [31]. It is worth noting that complex traumas like CSA often lead to ‘complex symptoms’ within CPTSD including low self-esteem which can discourage disclosure of abuse [32]. Despite growing literature globally, future research requires us to understand the barriers and facilitators of disclosures of CSA by adult survivors in the South Asian clinical settings.

Help-seeking for processing traumatic memories is limited by an inadequate understanding of trauma and its impact on mental health. Our study suggests that trauma-affected people prioritise seeking support for current symptoms causing functional impairment over processing their traumatic past. Mental and sexual health education in South Asian countries is limited [33] which may restrict survivors’ ability to articulate traumatic experiences like CSA. There is a pressing need to increase mental and sexual health awareness in a culturally appropriate manner. Future research could assess whether including mental and sexual health evaluations within general health evaluations could help identify sexual traumas and their impact.

The professionals commented that their clients prioritized a quick recovery over a complete recovery. Socioeconomic issues linked to inflation and poverty in the region and political conditions associated with communal riots, migration and structural stigma in South Asian countries [34, 35] would take precedence over multiple visits to a mental health professional.

Although our findings suggest that future research needs to aim at short-term treatments, the current evidence in the UK recommends psychological interventions lasting 8–12 sessions or more if needed [19]. There is limited evidence on single-session treatments for PTSD and other trauma-affected people following a single traumatic incident [36, 37]. Hence, before aiming for single-session or brief treatments, we propose conducting qualitative studies with adult CSA survivors to explore their views on the treatment needs and possible length of treatment. Then, conducting a feasibility trial would substantiate if we could proceed with the newly developed treatment informed by survivors’ views [38].

None of the mental health professionals in our study mentioned acute stress reactions while discussing any of their trauma-affected clients and instead diagnosed trauma survivors with other common and severe mental disorders. Before seeking professional help, trauma survivors, specifically adult CSA survivors, may seek informal support [39]. South Asians residing in the UK and the USA have reported structural stigma and culturally specific factors like subordination, encouraging informal support over formal ones [40]. Understanding treatment and support needs of adult CSA survivors in South Asia and the facilitators and barriers in seeking formal and informal sources of support warrants exploration.

We found that barring CSA, other forms of developmental trauma were not perceived by our participants’ clients as abuse. Notably, physical violence is the most reported form of violence against children in South Asia [41]. Parenting in South Asian culture has been labelled “authoritarian” in that cultural expectations require children to be obedient and follow a social hierarchy, and an increased need of the child to respond to parents’ demands [42, 43]. Considering that these are normative parenting styles in this region, critical comments and physical punishments to discipline the child could be blurring the boundaries between the accepted cultural norms of parenting and what is deemed to be physical abuse in non-South Asian cultures [44]. Various reports and articles have recommended increasing awareness about neglect and physical abuse in South Asia [41, 45, 46]. There is a need to include awareness about these other forms of abuse and neglect in childhood while endeavouring to increase awareness about the impacts of CSA in adulthood.

### Establishing culturally sensitive, specialist services and training

Psychological help was voiced by the professionals as the most relevant formal support for trauma affected people, including CSA survivors. In the UK and the USA, treatment guidelines for PTSD and emerging evidence for CPTSD also recommend CBT and other psychotherapeutic approaches as first line interventions [19, 47] Whilst psychological treatments were unanimously recommended by the professionals in this study, there were varied views on the need to process trauma memories, despite this being considered a key component of PTSD/CPTSD treatment in Western contexts [48]. Avoiding ‘unpleasant’ distressing memories and thoughts as an outcome of exposure to trauma could also be related to resistance to trauma memory processing by the survivors [49]. Future work could reflect on the efficacy of processing trauma memories and phase-based treatment with trauma survivors, specifically adult CSA survivors in South Asian countries.

A significant finding was the professionals’ and stakeholders’ systemic approach to the treatment of trauma affected people, specifically adult CSA survivors. In a systematic review including 31 studies with racially and ethnically diverse samples on systemic interventions for exposure to traumatic events, [50] found strong evidence for youth-caregiver, group and couple interventions to reduce PTSD and related symptoms. Specifically for adult CSA survivors, interpersonal relationship difficulties in adulthood would mean a systemic approach to the treatment could be beneficial [51].

One Stop Centres for trauma-affected people cited by Nepalese clinicians emerged as an example of centralised services for gender-based violence. Such centres have been set up in other South Asian countries too, including Bangladesh, India, Maldives and Sri Lanka [52–55].

Current lack of evidence on effective treatments for people affected by trauma, specifically adult CSA survivors, in South Asia warrants attention [5]. Compatibility between trauma affected clients’ cultural values and adaptations of evidence-based treatments [56] is essential for offering legible services to trauma survivors. Despite culturally adapting interventions being an “ethical responsibility” [57], the paucity of resources and trained researchers could be creating obstacles to such adaptations for trauma survivors in South Asian countries. A recent briefing paper by International Association for Traumatic Stress Studies for treating trauma affected people in LMICs (including South Asian countries) recommends cultural adaptations and research being led or co-led by LMIC researchers [58]. In our study, all professionals proposed adapting the current trauma focused treatments for use in their countries and according to client needs.

The current ICD-11 diagnostic criteria for PTSD/CPTSD acknowledges the cultural variation in the presentation of symptoms [15]. Cultural variation specifically exists in the disturbances in self-organisation symptoms but lacks further supporting evidence [59]. Due to the scarcity of evidence on CSA, developing an understanding of what constitutes psychological trauma in South Asia would be foundational to furthering treatments for trauma-affected people in the region. Future research into the clinical presentation would be beneficial to inform treatment and support services.

Our findings suggest that mental health research and sectors in South Asia are under-resourced. A recent study identified building research capacity in South Asia as one of the priorities to promote mental health [60]. Our findings suggest licensure and legal requirements for practitioners in South Asia are still unclear. This could risk the retention of currently qualified professionals within South Asian countries, further weakening the services. In addition, South Asian population experiences multiple catastrophic events [31]. Lacking an understanding of the culture-specific needs of trauma survivors, specifically adult CSA survivors and systemic issues in this region warrants an evidence base for treatment that may not be completely independent of Euro-centric literature on psychological trauma, but which is developed or refined from within these countries.

### Strengths and limitations

This is the first study, to the best of our knowledge, which explores the treatment and support needs of trauma-affected people, especially adult CSA survivors in South Asia, from the perspective of mental health professionals and key stakeholders. Further, we included professionals from six of the eight South Asian countries, who had varied qualifications and were at different career stages. The participants were practicing across Government and private institutes in diverse geographical regions, treating clients with varied socioeconomic statuses and from different communities. Although most participants were female, we did include four male and one gender fluid participant. Our research team was also diverse and included members from India, the UK and Europe, at different career stages with experience in trauma research and/or qualitative research. The interviews were conducted by an Indian clinician and researcher who was able to converse in Hindi as well as English.

Our study nevertheless has limitations. Whilst we included a diverse range of mental health professionals, some participants had limited experience of working with trauma affected people. Despite the best attempts of the research team to contact Bhutanese and Maldivian professionals, we were unable to recruit participants from these two countries, who continue to be underrepresented in mental health research. We were also only able to include two key stakeholders. A crucial perspective which is not heard in this study is that of trauma survivors themselves. This is an important next step in research in this field.

### Implications for practice, research and policy

Practitioners, both clinical and non-clinical, in South Asia need to be trained to ask their clients about any history of trauma, especially CSA. Setting up multidisciplinary specialist teams or specialist centres could improve the management of treatments for trauma survivors, specifically adult CSA survivors. Clinicians could include sexual health literacy in psychoeducation and stakeholders in their awareness programmes and individual sessions if they suspect a CSA history. Finally, trauma survivors could be on both sides of the table; ensuring sensitive peer supervision could be instrumental in supporting practitioners who may be survivors themselves. Clinical supervisors need to develop a model of support for professionals and researchers working with trauma affected people.

For researchers, there are dual implications for survivors and professionals. There is a need to identify the mental health and support needs of trauma, specifically CSA, survivors; explore key facilitators and barriers in their help seeking; and develop culturally tailored interventions to best meet the identified needs of survivors. Future studies should also explore the psychological impact potentially on the trauma practitioners and researchers.

Policy makers need to improve the regulation of mental health professionals, their training and licensure requirements and increase the number of clinical training programmes in mental health in South Asia. This would need to include specialist trauma treatment training and allocating funds to set up services for trauma survivors but specifically for adult CSA survivors. There is a need for advocating policy development and implementation aimed at improving reporting mechanisms of CSA. In addition, enhancing legal protection for someone reporting CSA could prevent further incidents of CSA. Considering that many CSA survivors may not seek clinical help, there is a need to fund support services for the survivors in the non-statutory sector. Finally, there is a need to create awareness among children and their guardians about mental health conditions and the nature and impacts of abuse on children. This would include accessible visual materials and credible sources of support.

## Conclusion

The mental health and support needs of trauma survivors, specifically adult CSA survivors, have been identified by practitioners in South Asian countries. However, offering appropriate, culturally tailored specialist services would require better training, improved funding and identifying systemic facilitators to support this vulnerable population. It is also imperative to explore the specific needs of adult CSA and other trauma survivors in the South Asian context.

## Supporting information

S1 Text Topic guide for interviews with mental health professionals. (DOCX)

S2 Text Topic guide for interviews with key stakeholders. (DOCX)
