# Supplementary material for: Mental health professionals and key stakeholder views on the treatment and support needs of trauma and adult survivors of childhood sexual abuse in South Asia
Source: PLOS Ment Health. 2024 Sep 13;1(4):e0000136. doi: 10.1371/journal.pmen.0000136 (PMC12798635; doi:10.1371/journal.pmen.0000136)
Supplement: S1 Text — (DOCX) [file pmen.0000136.s001.docx]

**S1 Topic guide for interviews with mental health professionals**

Initial questions about their role and work with people who have experienced trauma and PTSD/CPTSD:

1. Can you tell me a bit about your role here?
2. What kind of issues do your clients come to you with?
3. What kinds of treatments do you offer to a client affected by trauma?
4. What are their common issues?
5. What are the types of traumatic incidents they report?
6. When do they bring it up?

Questions about working with people who experienced interpersonal and/or collective trauma

1. How do you approach their treatment if they report a history of interpersonal trauma like child abuse, neglect?
2. What kinds of treatments are offered?
3. How do you formulate their issues?
4. Do you have patients who report any collective trauma like disaster, war, occupational?
5. What kinds of treatments are offered?
6. How do you formulate their issues?

Is there something you would like to add which I did not ask you today?
