# Supplementary material for: Mental health professionals and key stakeholder views on the treatment and support needs of trauma and adult survivors of childhood sexual abuse in South Asia
Source: PLOS Ment Health. 2024 Sep 13;1(4):e0000136. doi: 10.1371/journal.pmen.0000136 (PMC12798635; doi:10.1371/journal.pmen.0000136)
Supplement: S2 Text — (DOCX) [file pmen.0000136.s002.docx]

**S2 Topic guide for interviews with key stakeholders**

Initial questions about their role and work with people who have experienced trauma and PTSD/CPTSD:

1. Can you tell me a bit about your role here?
2. What kind of issues do people approach you with?
3. What kinds of programmes do you offer to a person affected by trauma?
4. What kind of programmes do you offer to a person diagnosed with PTSD/CPTSD?
5. What are their common issues?
6. What are the types of traumatic incidents they report?
7. What do you think they need support with?

Questions about working with people who experienced interpersonal and/or collective trauma

1. How do you approach the discussion if they report a history of interpersonal trauma like child abuse, neglect?
2. Do you have people who come to you with any collective trauma like disaster, war, occupational?

Is there something you would like to add which I did not ask you today?
